# Use of Consumer Wearables in Health Research: Issues and Considerations

**DOI:** 10.2196/52444

**Published:** 2023-11-21

**Authors:** Rosie Dobson, Melanie Stowell, Jim Warren, Taria Tane, Lin Ni, Yulong Gu, Judith McCool, Robyn Whittaker

**Affiliations:** 1 School of Population Health University of Auckland Auckland New Zealand; 2 Institute for Innovation and Improvement Te Whatu Ora Waitematā Auckland New Zealand; 3 School of Computer Science University of Auckland Auckland New Zealand; 4 School of Health Sciences Stockton University Galloway, NJ United States

**Keywords:** wearable device, wearable, wearables, inclusion, inclusive, inclusivity, data quality, consumer wearables, sensors, digital health, mental health, ethics, ethic, ethical, privacy, security, viewpoint, digital divide, data privacy, health information management, data science, data collection

## Abstract

As wearable devices, which allow individuals to track and self-manage their health, become more ubiquitous, the opportunities are growing for researchers to use these sensors within interventions and for data collection. They offer access to data that are captured continuously, passively, and pragmatically with minimal user burden, providing huge advantages for health research. However, the growth in their use must be coupled with consideration of their potential limitations, in particular, digital inclusion, data availability, privacy, ethics of third-party involvement, data quality, and potential for adverse consequences. In this paper, we discuss these issues and strategies used to prevent or mitigate them and recommendations for researchers using wearables as part of interventions or for data collection.

## Introduction

The use of wearable devices, which permit individuals to track and self-manage their health, is rising as health care becomes increasingly digitalized [1,2]. Wearables collect physiological data relevant to the diagnosis, monitoring, prediction, and treatment of a range of conditions including cardiovascular, functional, and mental health through noninvasive measurement of physiological information in real time [2-5]. The available sensors include medical-grade devices requiring governmental approval, such as remote patient monitors, as well as consumer-grade wellness devices, such as smartwatches and fitness trackers worn on the body [6].

With wearables becoming more ubiquitous, the opportunity has emerged for researchers to analyze rich sensor data that are captured continuously, passively, and pragmatically with minimal user burden [7-9]. For instance, several reviews of the research regarding wearable sensors for mental health management have highlighted the potential of wearables as a useful tool for self-care (eg, episode detection and intervention) and for facilitating remote psychiatric monitoring [4,10-12]. The use of this approach was perhaps most apparent during the COVID-19 pandemic, when remote data collection methods became necessary, and wearables allowed web-based studies with large cohorts to continue despite government-mandated restrictions on in-person contact [7]. The potential benefits of these devices extend to the value of enabling data collection within everyday settings away from the distorted conditions of laboratory or clinical environments and to the potential inclusion of populations in research for which research facilities have not been accessible.

Despite these advantages, a need has been identified for more robust research to establish evidence about the feasibility, acceptability, and efficacy of health interventions leveraging wearables [4,7,13]. In particular, the generalizability of study outcomes has come under question due to homogenous study samples [1,5] and a focus on high-income contexts [7]. Concerns have also been raised about consumer privacy [12], data reliability [13], and digital inclusion in wearables research [5].

Through our research using consumer-grade wearables in the identification and management of anxiety in young adults, we have identified a number of challenges and issues including digital inclusion, the availability of data, privacy, ethics of third-party involvement, data quality, and potential for adverse consequences. In this paper, we discuss each of these issues and describe strategies used in other studies to prevent or mitigate them. We also provide recommendations for other researchers using wearables as part of interventions or for data collection.

## Digital Inclusion

We have been conducting research to investigate how sensors can be used to identify the early changes associated with an anxiety episode to provide the opportunity for earlier brief intervention. Early work has involved the labeling of data collected through consumer-grade wearables with self-reported anxiety levels, as well as assessing the acceptability and feasibility of sensors for use in anxiety interventions. A research charter we developed at the research commencement outlined ethical and professional values and principles to underpin the work carried out. A key principle outlined in the research charter is equity, where interventions are designed to reduce health inequities, and that population groups that experience the greatest health inequities are prioritized at every stage of the research process. Furthermore, there should be no barriers to participate in the research (eg, research costs and requiring device access) and the resultant interventions or technologies, if effective, should be easily scalable across the population, including in low socioeconomic groups. This means that when using wearable sensors in research studies, we need to choose devices that are low-cost and easily accessible to consumers. However, using commonly available consumer wearables raises complexities related to access to data, privacy and security, and secondary use of data.

## Data Access and Privacy

In our initial assessment of the currently available consumer wearables which included 10 brands and 31 models, we sought to identify a wearable at an accessible price point that also allowed end users to access the raw data relevant to our study, particularly heart rate variability (HRV) and electrocardiogram. At the time of our assessment, which was completed in April 2021, only the Polar H10 and Fitbit Sense (Fitbit) were deemed able to provide access to the specific data that we needed. However, accessing the full raw data still required us to download a third-party data logging app (in the case of Polar H10) or register for a Fitbit Premium membership, which involves a paid subscription. The question of data availability and user access to raw data has been highlighted as an important consideration for researchers but one that has not been adequately discussed in individual studies [7].

We also observed that the algorithms used by vendors to provide health reports are often unique, dynamic, and not disclosed to consumers, thus making it difficult for researchers to understand any data discrepancies or potential inaccuracies where multiple devices are being tested. This finding has been noted elsewhere [6,7,14].

Where raw data from wearables are available to consumers, privacy issues for consumers and researchers have been noted [15,16]. While in research we can obtain participants’ informed consent to collect and use their wearable data, many companies present privacy policies that often go unread or poorly understood by consumers, leaving them unaware of how their health data may be stored, used, or shared [17]. As consumer wearables are not regulated in the same way as medical-grade devices and data protection laws vary by location, company practices have been criticized as inadequate in informing and protecting consumers whose data are being collected and shared with third parties [6]. This has been highlighted as a barrier to user adoption of wearables [12].

## Ethics of Endorsing Wearable Companies

Researchers’ use and possible recommendation of wearables for health screening and management may be perceived as a direct or indirect endorsement of the developing company’s practices. The use of wearables in research may lead to commercial gains for the company and increased user exposure to marketing, which researchers have little ability to control. Further, there is no control over companies’ use of individual data for secondary purposes, which users may not consider when agreeing to participate in studies using the devices. Among other issues surrounding the use of data for secondary purposes, this could potentially be an issue in upholding the rights of indigenous data sovereignty.

## Data Quality

As previously mentioned, in this study, we sought to identify consumer wearables that track and provide user access to user HRV data as an in-the-moment indicator of heightened stress [13,18]. In our initial assessment of wearables on the market, conducted in early 2021, it appeared that many available wearables collected HRV data. However, upon further investigation, through data from our field-testing study, we learned that many of the common wrist or chest-worn sensors on the market did not collect HRV continuously, provide easy consumer access to the raw data, or provide adequate contextual information. This finding has been echoed by others [18] and would prevent us from reaching our goal of developing an in-the-moment intervention to support young adults’ times of heightened stress using existing wearables [4]. Other metrics commonly collected by wearables such as heart rate and step count have thus far demonstrated to be less accurate in predicting stressful episodes in our experience, evoking the lesson that “more” data are not necessarily “better” if they do not speak to the outcome of interest. Full results of our field-testing study are forthcoming and will be published.

Since beginning our field testing using the Polar H10 and Fitbit Sense, more issues related to data quality have been identified, including data “noise” such as passive movements being inaccurately logged as true physical activity and Bluetooth connectivity issues resulting in missing data. Others testing wearables for the detection of atrial fibrillation have observed a significant potential for false-positive diagnosis and have expressed concern about patients receiving unwarranted anticoagulant treatment [19]. Moreover, in addition to the cost of the wearable itself, recording and transferring data for in-the-moment interventions requires individuals’ access to devices with continuous Bluetooth or cellular data connectivity, which presents challenges from an equity perspective. Others have similarly observed that successful wearable use requires a high degree of economic status and digital literacy, which may pose particular barriers for minoritized populations such as older adults and those on low income [1,5]. For optimal data quality, we need to ensure adequate training for users alongside ensuring that those analyzing and interpreting the data are aware of the possible sources of error in order not to draw false conclusions.

## Potential Adverse Consequences of Wearables

The drawbacks of increased digitalization and reliance on technology must also be considered when using wearables for health purposes. The impacts of increased digital connectivity on individuals, particularly young people, are unclear but frequently suspected to be negative [20,21]. It is, therefore, worth considering that wearables, especially those connected to social media, may negatively impact people’s mental health and feelings of isolation [22]. Moreover, continuous feedback and alerts about one’s physiological state may put some individuals at risk for pathologic self-monitoring [23-25] or compensatory behaviors [26]. Are wearables therefore part of the “anxiety problem” and is it appropriate to use them to help? A subsequent phase of our study will explore the acceptability of using wearables for mental health purposes among young adults to understand any negative consequences of continuous wearable use. Previous research has shown that people’s engagement with consumer wearables is highly individual [27], highlighting not only the complexity of this issue but also that there may not be a straightforward answer to this question. If we are going to use these, we need to consider how we can ensure transparency and adequate education about the extent of the monitoring and user control of this, and the benefits and risks of the wearables.

## Discussion and Recommendations

As robust research about the efficacy and acceptability of consumer wearables continues to emerge, we echo recommendations for policies and legislation to protect consumer privacy and for companies to be more transparent about their algorithms and data processing procedures [6]. Data trusts, in which all stakeholders (eg, vendors, researchers, clinicians, and consumers) engage in a legally binding data management framework, have been suggested as a means of increasing consumers’ access to their data while continuing to provide beneficial information to other vested parties [7,28]. We also reiterate recommendations that researchers carefully consider the availability and access to wearable user data as part of their study planning [7].

In the meantime, researchers may seek to develop their own methods of capturing the data they require, such as bespoke applications to which the raw wearable data, if available, can be transferred and processed to meet the needs of the study [29]. Nonetheless, researchers must consider that wearable companies’ practices and ethos may change at any time, with implications for the products they sell and customer treatment.

Wearables are continuously being updated and improved by developers to enhance device performance and capabilities. With minimal governmental oversight of consumer-grade wearables, it is critical that researchers continue to test devices for accuracy and reliability and to hold companies accountable for their claims. Devices at accessible price points, while maintaining accuracy and data quality, must also be a priority for developers if wearables are to become an equitable solution in individual health management.

As the use of wearables for health monitoring increases, establishing evidence for the possible negative impacts of wearables must be prioritized. Researchers may consider testing the efficacy and acceptability of wearable use in which the visibility of health metrics is not automatically and continuously fed to users but rather collected passively and only made available to users when explicitly sought. In-the-moment interventions and support may therefore still be implemented using data being processed in the background while minimizing the risk of disruption and stress to users and therefore increasing adherence.

## Conclusions

Despite their potential to improve the way we track and manage individuals’ health, the use of consumer wearables comes with a unique set of challenges for researchers which must be carefully weighed. It is important that associated challenges, such as those discussed in this paper, are reported alongside any positive outcomes. Without this, government policies and company practices cannot effectively adapt to protect digital health equity, individuals’ privacy and access to data, and to ensure that wearables provide the desired level of information and support to their users.

## Abbreviations

HRV: heart rate variability
